# Hair cortisol varies with season and lifestyle and relates to human interactions in German shepherd dogs

**DOI:** 10.1038/srep19631

**Published:** 2016-01-21

**Authors:** Lina S. V. Roth, Åshild Faresjö, Elvar Theodorsson, Per Jensen

**Affiliations:** 1 Linköping University, IFM Biology, 581 85 Linköping, Sweden; 2 Linköping University, Department of Medical and Health Sciences, 581 85 Linköping, Sweden; 3 Linköping University, Department of Clinical and Experimental Medicine, 581 85 Linköping, Sweden

## Abstract

It is challenging to measure long-term endocrine stress responses in animals. We investigated whether cortisol extracted from dog hair reflected the levels of activity and stress long-term, during weeks and months. Hair samples from in total 59 German shepherds were analysed. Samples for measuring cortisol concentrations were collected at three occasions and we complemented the data with individual scores from the Canine Behavioural Assessment and Research Questionnaire (C-BARQ). Generalised linear mixed model (GLMM) results showed that hair cortisol varied with season and lifestyle: competition dogs had higher levels than companion, and professional working dogs, and levels were higher in January than in May and September. In addition, a positive correlation was found between the cortisol levels and the C-BARQ score for stranger-directed aggression (r = 0.31, P = 0.036). Interestingly, the factor “playing often with the dog” (r = −0.34, P = 0.019) and “reward with a treat/toy when the dog behaves correctly” (r = −0.37, P = 0.010) correlated negatively with cortisol levels, suggesting that positive human interactions reduce stress. In conclusion, hair cortisol is a promising method for revealing the activity of the HPA-axis over a longer period of time, and human interactions influence the cortisol level in dogs.

Cortisol secretion is the result of the activation of the hypothalamic pituitary adrenocortical (HPA) axis, and plays a crucial part in the body’s response to different kinds of biological stress^1^,^2^. In dogs, increased cortisol level can indicate acute stress from sudden fearful stimuli and is possible to determine in real time with blood and saliva measurements^3^,^4^. It has also been shown that a dog park visit is associated with increased salivary cortisol levels while a normal walk does not necessarily change the dog’s cortisol level^5^. In addition, results suggest habituation to novel and potential stressful situations, since the cortisol levels were negatively correlated with dog park visit frequency^5^. Similar negative correlation was found with number of days a dog is staying in an animal shelter, suggesting habituation to the situation^6^.

Since cortisol levels in blood and saliva correlate well^7^, non-invasive saliva sampling has become a useful method to avoid additional stress due to sampling. Nevertheless, both cortisol in blood and saliva reflect a momentary measurement in real time and to measure long-term cortisol secretion, indicating possible long-term stress, multiple samples are needed. Cortisol has also been shown to have a circadian rhythm both in dogs and humans^8^,^9^ which makes repeated measurements from different times of the day imprecise.

Therefore, a promising non-invasive method to study prolonged changes in the HPA-axis activity is to measure cortisol incorporated in hair^10^,^11^,^12^,^13^,^14^,^15^. Hair cortisol has been extensively studied during the last years and correlates positively with cortisol levels in both saliva^13^ and faeces^16^ of dogs. In addition, rhesus macaques exposed to prolonged stress^14^ and lynxes that were weekly injected with ACTH^17^ (corticotrophin that results in secretion of cortisol from the adrenals) all showed clear increase in hair cortisol. Correspondingly, dogs with hypercortisolism have higher hair cortisol values than healthy dogs^12^ and studies on humans show increased hair cortisol levels in people with chronic pain^18^, in unemployed or depressed people^19^,^20^ and after major life events^21^. Hence, hair cortisol is a promising indicator of long term HPA-axis activity.

However, even though hair cortisol reveals long-term cortisol secretion and can be an indicator of chronic stress, we also need to consider possible seasonal variations. From salivary cortisol studies it is shown that levels vary between seasons with high levels during the winter and low during the summer^9^,^22^. It is likely that this is the case also in hair cortisol even though, to our knowledge, there is no study confirming this.

The aim of this study is to investigate potential relationships between hair cortisol and general lifestyle patterns in dogs and also to reveal possible differences in cortisol secretion throughout the year. To exclude possible breed or size differences, which may influence cortisol levels^23^, we focused on one breed, the German shepherd. Hair was sampled at three occasions from the same dogs and we also analysed different types of hair (wool and guard hair) from two body sites (chest and neck) in both companion, competition and professional working dogs. We complement the study with results from the validated Canine Behavioral Assessment and Research Questionnaire (C-BARQ)^24^ and additional questionnaires to investigate possible causes of differences in hair cortisol levels.

## Material and Methods

### Animals

In total 59 German shepherd dogs were used in this project (Table 1, Supplementary 1). We sampled 47 in January, 45 in May and 38 in September. Hence, 38 dogs were sampled at all three occasions and these repeatedly sampled dogs were grouped according to owner opinion into three lifestyle groups: companion, competition or professional working dogs. Note that additional 12 German shepherds were sampled in September and were not used in the analysis of seasonal and lifestyle effects but only for correlations with questionnaire results. The additional dogs were therefor not grouped according to lifestyle. Most of the dogs were privately owned with the exception of those from the Police (N = 6) and Armed forces (N = 8) and all dogs were recruited through social media or personal contacts. One male dog was later excluded from all cortisol analyses because of extreme, possibly pathological, cortisol levels (>500 pg/mg). All experiments in this paper were conducted in line with ethical approval from the regional ethical committee for animal experiments in Linköping, Sweden (Permit number: 51–13).

### Hair sampling

**Figure 1:**
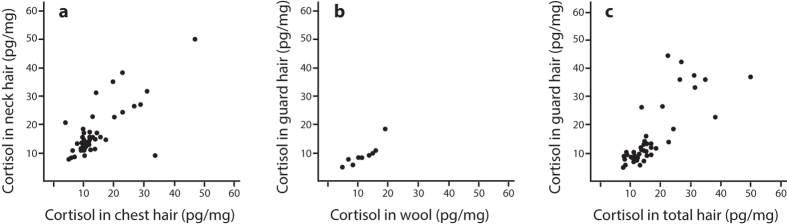
Correlations between hair cortisol (pg/mg) in neck and chest hair (**a**), in wool and guard hair (**b**), and in total hair and the separated guard hair (**c**), all from the January sampling occasion. Three values above 60 pg/mg in (**a**,**c**) have been omitted from the figures for clarity; (**a)** (neck vs chest): 173 vs 60 pg/mg, 124.4 vs 101.2 pg/mg and 166.1 vs 372.8 pg/mg; (**c)** (guard hair vs total hair): 156.0 vs 173.4 pg/mg, 75.3 vs 124.4 pg/mg and 168.5 vs 166.1 pg/mg).

Hair samples were obtained by cutting approximately 0.5 g hair with a pair of scissors as close to the skin as possible without injuring the dog. In January hair was sampled from both chest and neck of the dogs, but since strong correlation was found in hair cortisol between the two sites (See Results; Fig. 1a) and to minimise possible stress and risk of injuring during sampling only neck hair was sampled in May and September. We also decided to focus on only the guard hair in consistency with other studies^25^. Even so the January hair samples were analysed as both total hair (wool and guard hair as obtained from the dog), and separately in guard hair only (See Results; Fig. 1c). In addition, the separated wool was analysed from 10 dogs (See Results; Fig. 1b).

### Hair preparation and cortisol extraction

Hair samples were stored in room temperature until preparation and cortisol extraction which was performed according to methods described in detail previously^21^,^26^. Briefly, 5–10 mg from each sample were cut into small pieces (<3 mm), frozen 2 min in liquid nitrogen and minced together with a steel ball using a Retch Tissue Lyser II in 2 min. Methanol (1 ml) was added to each tube and the samples extracted for at least 10 hours on a moving board. 0.8 ml of the methanol supernatant was pipetted off and lyophilized using a Savant Speed Vac Plus SC210A and the samples were dissolved in radioimmunoassay buffer and analysed as described by Morelius *et al.*^26^. Hair samples of 5 mg or more were needed for maintaining a total inter-assay coefficient of variation below 8% for hair extraction and measurement of cortisol by the radioimmunoassay. The intra-assay coefficient of variation for the radioimmunoassay itself was 7% at 10 nmol/L. Taking the binding of cortisol as 100%, the antiserum cross-reacts 137% with 5α-dihydroxycortisol, 35,9% with 21-deoxycortisol, 35,9% with prednisolone but less than 1% with endogenous steroids. For a detailed description of the method, see Karlén *et al.*^21^.

### Questionnaires and C-BARQ

At each sampling occasion for the 47 dogs sampled in January, May and September, the owners were asked to answer a simple questionnaire. Here, the owners described the main lifestyle of their dog (companion, competition or professional working dog), and the dogs were later grouped according to those answers. Other questions were about background information (name, age, sex, castration), medication, home environment (other animals/dogs or kids in the household). Most questions generated answers with little variation and were not included in further statistical analyses. Furthermore, questions were asked about the frequency of organised training sessions (scale 0–3 where 0 = 0 sessions, 1 = 1–6 sessions, 2 = 7–15 sessions, 3 = 16 or more sessions) and competition frequency (scale 0–3 where 0 = 0 occasions, 1 = 1–3 occasions, 2 = 4–8 occasions, 3 = 9 or more competition occasions) during the last three months and these variables were later analysed for possible correlation with the hair cortisol levels. The owners also included information about what kind of training they performed. More than half of the companion dogs (10 out of 17 dogs) performed different forms of obedience and tracking training while the majority of the competition dogs (12 out of 16 dogs) were trained in IPO (Internationale Prüfungs-Ordnung) which includes both tracking, obedience and high level of protection work.

At the last sampling occasion (September), the owners were asked to complete a C-BARQ questionnaire, which is a validated and frequently used instrument to collect data about dog behaviour and personality in everyday life^24^. All but three completed the questionnaire. The C-BARQ included 105 questions or statements, where the owners rated their dogs on a scale from 0–4 (0 = never, 1 = seldom, 2 = sometimes, 3 = usually, and 4 = always). The scores were later added to form 13 behavioural categories, according to the standards of the test, and these categories were analysed for possible correlations with the hair cortisol levels. An additional 17 scaled questions (0–4, where 0 translates into “do not agree” and 4 into “totally agree”) about play, reward, corrections, cooperation, focus ability, and training were answered in connection with the C-BARQ.

### Statistical analyses

All statistics were performed in the software SPSS (version 23, IBM).

Due to non-parametric distribution of the hair cortisol levels in the neck and chest hair samples and in the wool and guard hair samples analysed in January, the Spearman’s nonparametric rank-order correlation was used. The Spearman’s nonparametric rank-order correlation was also used for all correlations between cortisol levels and C-BARQ scores and the additional 17 questions.

Generalised linear mixed models (GLMM) with cortisol level (pg/mg) as Fixed target and Gamma log as probability distribution were used to analyse effects of season, using repeated measures. Time (three levels: January, May, September) and lifestyle (three levels: companion, competition or professional working dog) were treated as fixed effects together with their interaction. Individual dogs were included as random effects in order to achieve as good model as possible according to Akaike’s Information Criterion (AIC). Training frequency, age and sex were also tested as fixed effects but were excluded based on AIC. Twelve dogs sampled only in September for which data on lifestyle was not available, were not included in the GLMM but used in correlation analyses between cortisol and questionnaire results.

Kruskal Wallis tests were used to investigate differences between companion, competition and working dogs for the C-BARQ scores due to the ordinal nature of data.

## Results

### Hair cortisol from different body sites and hair types

Cortisol levels from the January chest hair correlated positively with the neck hair (Fig. 1a, total hair was analysed, i.e. wool and guard hair together, r = 0.70, P = 0.001, N = 46). In addition, separating ten of the hair samples from the neck into wool and guard hair revealed a strong positive correlation of cortisol levels in the two hair types (Fig. 1b, r = 0.99, P = 0.001). Similarly, we found a significant positive correlation between the hair cortisol level in total hair and in the separated guard hairs, both sampled from the neck of the dog in January (Fig. 1c, r = 0.85, P = 0.001, N = 46).

### Cortisol variation with season and lifestyle

**Figure 2:**
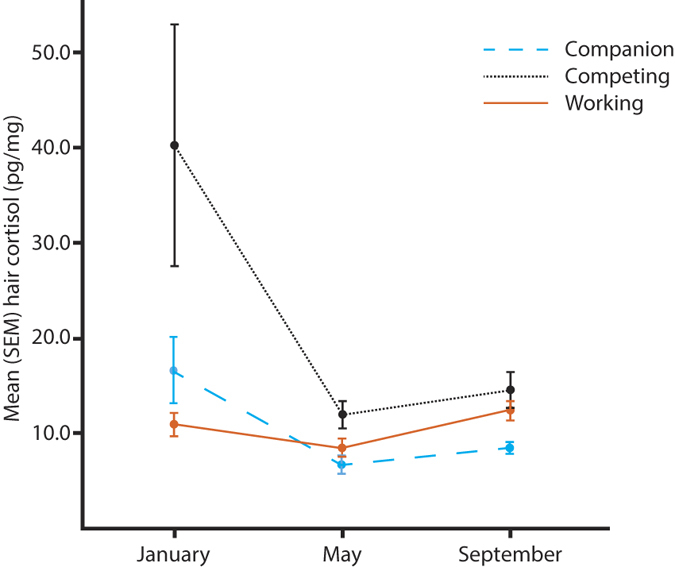
Mean hair cortisol level (pg/mg) in January, May and September for companion, competition and working German shepherds. Standard Error of Mean (SEM) is shown with error bars.

Both time of the year, lifestyle and the interaction between the two showed significant effects on the hair cortisol level (Table 2). Thus, there was a seasonal effect and the cortisol level also depended on the lifestyle, where the competition dogs (mainly IPO trained dogs) had higher cortisol levels than both companion and working dogs (Fig. 2). However, no correlation between cortisol and training frequency, as assessed by the owners themselves, was found (January: r = −0.27, P = 0.40, May: r = 0.03, P = 0.87, September: r = 0.05, P = 0.77) and no correlation was found with age of the dog (January: r = −0.18, P = 0.24, May: r = 0.13, P = 0.41, September: r = 0.04, P = 0.84).

### Cortisol correlations with owner questionnaires

A positive correlation was found between cortisol level and the C-BARQ score “stranger-directed aggression” (r = 0.31, P = 0.036) for the dogs sampled in September (N = 46) and there was also a negative correlation between cortisol and the C-BARQ score “Chasing” (r = −0.38, P = 0.009). Interestingly, correlations between cortisol levels and the responses to additional questions included in September questionnaires revealed a negative correlation with the scores on “the purpose of the dog is to have a nice companion dog” (r = −0.32, P = 0.029), “play often with the dog” (r = −0.34, P = 0.019), and “reward with a treat/toy when the dog behaves correctly” (r = −0.37, P = 0.010). No other scores on CBARQ categories or additional questions were significantly correlated with cortisol levels.

### C-BARQ differences between companion, competition and working dogs.

Analyses of the C-BARQ scores in September for the companion, competition and working dogs showed significant differences for “dog-directed aggression” (higher in working dogs; P = 0.04) and “non-social fear” (higher in companion dogs; P = 0.004, Table 3). There were no other significant differences between the groups of dogs with respect to questionnaire data.

## Discussion

To our knowledge this is the first study that has investigated seasonal variation and variations related to lifestyle in long-term cortisol secretion in dogs. The results from the study show that both season and lifestyle significantly influence the hair cortisol levels.

Earlier studies on other species have suggested that there are differences in hair cortisol depending on body location^17^,^25^,^27^. However, in our study, the levels of cortisol correlated strongly between different body parts, so the method appears highly reliable for long-term assessments over time in the same dogs.

Investigating the cortisol levels in wool and guard hair separately revealed high correlation and no significant difference between the hair types. Hence, the method appears to be quite insensitive with respect to which parts of the fur that is used for hormone extraction. However, differences have been reported in other species^25^, so we continued with analysing only guard hair to make the results as comparable as possible between individuals, between breeds in future studies, and possibly also with other species with different hair compositions. Further studies and standardisations of the sampling method are likely to guide future comparative studies.

Our results suggest that seasonal variations need to be considered in cortisol studies. We found highest hair cortisol values in January. Since the hair grows gradually^28^, these levels reflect the dogs’ secretion in the preceding late fall and early winter in Sweden. The results therefore indicate an increased stress or activity level during this period, particularly in competition dogs. In a recent study on hair cortisol in foals during three consecutive foaling seasons (January to July) no effects were found of either temperature, or day length^29^. However, in polar bears hair cortisol was shown to heavily depend on fluctuations in climate and ice cover^30^, and as mentioned before some saliva cortisol studies suggest high cortisol levels during the winter^9^,^22^. Hence, possible seasonal variations need to be considered when sampling and comparing cortisol levels irrespectively of method used.

Different lifestyles and human demands on dogs might affect their activity pattern and HPA-axis activity. We found that competition dogs had higher hair cortisol levels than both companion and working dogs and this was especially obvious in January. Since a few millimetres proximal to the hair follicle were not included in hair sample (some part is even below the skin) the novel situation of obtaining hair (which lasted less than a minute) could not have induced this increase. The cortisol increase could possibly be related to variations in the amount of training since competition dogs usually train less during the winter and more during spring and in connection to competition season. A sudden decrease in training and competing during the late fall and winter, which is a common situation for many competition dogs, could possibly be experienced as an unpredictable stress experience^2^. Or , the dogs might simply be less exercised after competition season which might induce restlessness. However, no correlation or interaction between cortisol and training frequency was found, but it should be noted that the information and estimation of the training intensity was relatively crude in our experiments and we have no data on general exercise. Other studies have found relationship between cortisol and training, for example, increased cortisol levels were found in a study on faecal cortisol during training activities in avalanche dogs^31^. Still, an interesting question for future studies is whether large unpredictable changes in activity level i.e. from a day of rest to an intensive training session could be perceived as unpredictable and stressful, unlike the situation for professional working dogs that are more or less active throughout the day or companion dogs where the contrasts between rest and walks or light training sessions is small.

An additional possibility is that the dogs used for competition are chosen because of certain personality traits that could be accompanied by high cortisol levels. For example, hair cortisol correlates positively with the personality trait “reactivity” in dogs^32^. The majority (75%) of the competition dogs in the present study were trained and competed in IPO (Internationale Prüfungs-Ordnung), which includes both tracking, obedience and high level of protection work, and thereby requires dogs with high levels of reactivity and assertiveness. It could also be that the IPO dogs are exposed to different training methods than other German shepherd dogs, but more studies are needed to evaluate this.

From the C-BARQ results we found a positive correlation between ”stranger-directed aggression” and cortisol levels, corroborating previous studies^33^. Since competition dogs showed higher cortisol levels, one possibility could have been that they would therefor also show more stranger-directed aggression, but in fact the C-BARQ results for companion, competition and working dogs only differed for “Dog-directed aggression” and “non-social fear” where competition dogs showed low scores. No significant difference between the groups was found for “stranger-directed aggression”. Hence, more studies are needed to elucidate the exact relationship between cortisol levels and different types of aggression and fear.

In addition, a negative correlation between the C-BARQ score for “chasing” and cortisol was found. The questions behind this score ask how the dog behaves towards e.g. cats, squirrels and birds and whether the dog is prone to chase them. It could possibly be that dogs that do not chase due to training generate a suppressed motivation of hunting behaviour, which might, if exposed to the situation frequently, increase the cortisol level. But this is highly speculative and needs more investigation.

Maybe not surprising but still welcome result is that a negative correlation was found between cortisol level and how often the owner played with their dog and also whether the owners used toy/treat when rewarding their dog. Both these results could reflect that friendly and encouraging relationships are related to less stress in the dogs. Play interactions including affectionate behaviour have earlier been shown to have a direct decreasing effect on cortisol levels in dogs^34^, and dogs treated with corticosteroids are also less playful^35^, which both are in line with our findings on long-term cortisol secretion. These results may be important for understanding the physiological consequences of different types of human-dog relationships.

## Conclusion

Hair cortisol is a reliable long-term assessment method in dogs and our results show high consistency between different body sites and hair types. There were seasonal fluctuations in cortisol levels and they also varied in relation to lifestyle. In addition, cortisol levels were negatively correlated with friendly human interactions and positively correlated with human-directed aggression.

## Additional Information

**How to cite this article**: Roth, L. S.V. *et al.* Hair cortisol varies with season and lifestyle and relates to human interactions in German shepherd dogs. *Sci. Rep.* **6**, 19631; doi: 10.1038/srep19631 (2016).

## Supplementary Material

Supplementary Information

## Figures and Tables

**Table 1:** Sex and age (in years) distribution of the analysed German shepherds (N = 59).

Lifestyle group	Total N	Females	Males	Age (mean ± SEM)
Companion	17	10 (0)	7 (2)	3.1 ± 0.7
Competition	16	9 (0)	7 (2)	3.6 ± 0.5
Working (including the excluded male)	14	4 (1)	10 (3)	4.8 ± 0.4
Additional unspecified dogs	12	8	4	2.2 ± 0.4

Castrated/sterilised dogs are shown in brackets. For 12 additional dogs (only sampled in September) no lifestyle group was determined and neutered status was not available.

**Table 2:** Generalised linear mixed model with cortisol level (pg/mg) as fixed target and time, lifestyle and the interaction Time*Lifestyle treated as fixed factors and individual dog treated as random effect (N = 46).

	F	df1	df2	Sign.
Corrected model	9.16	8	118	0.001
Time (Jan, May, Sep)	18.59	2	118	0.001
Lifestyle (companion, competition, working)	11.04	2	118	0.001
Time * Lifestyle	2.93	4	118	0.024

**Table 3:** C-BARQ scores “Dog-directed aggression” and “Non-social fear” for companion (N = 14), competition (N = 11) and working dogs (N = 10).

C-BARQ score	Dog group	Median	Mean Rank	Mean	SEM
Dog-directed aggression	Companion	1.00	15.21	1.21	0.35
	Competition	1.00	15.32	1.11	0.17
	Working	2.25	24.85	2.10	0.26
Non-social fear					
	Companion	0.33	23.00	0.35	0.07
	Competition	0.00	10.41	0.03	0.03
	Working	0.17	19.35	0.22	0.06
